# Supplementary material for: Molecular Characterization of miRNAs in Myzus persicae Carrying Brassica Yellows Virus
Source: Biology (Basel). 2024 Nov 18;13(11):941. doi: 10.3390/biology13110941 (PMC11591976; doi:10.3390/biology13110941)
Supplement: Supplementary file 1 [file biology-13-00941-s001.zip › Data S3. Structures of novel miRNAs under treatment 2/novel_204_novel_204.pdf]

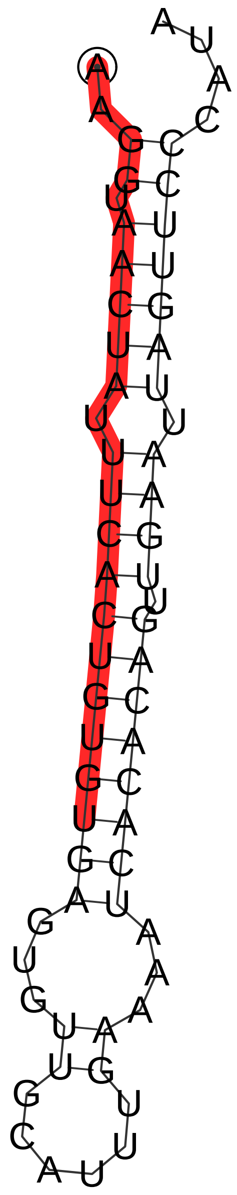

Secondary structure for 'novel_204'
